# Strabismus and Pediatric Psychiatric Illness: A Literature Review

**DOI:** 10.3390/children10040607

**Published:** 2023-03-23

**Authors:** Tiffany L. Huang, Stacy L. Pineles

**Affiliations:** Department of Ophthalmology, Stein Eye Institute, University of California, Los Angeles, CA 90025, USA

**Keywords:** strabismus, psychiatric illness, mood disorders, social stigmata

## Abstract

Purpose: This literature review aims to investigate the potential association between strabismus and mental illness among children. Materials: The search was conducted in the PubMed and Google Scholar databases using a wide range of search terms related to strabismus, mental disorders, psychiatric illness, childhood, and adolescence. Results: Eleven published studies were included in this review. The findings from this review suggest an association between strabismus and mental illness. Negative attitudes and social bias against children with strabismus were also noted. Conclusions: These findings should alert healthcare providers to counsel children and their caregivers regarding the risk for mood disorders in children with strabismus and to consider mental health screening and referral as needed.

## 1. Introduction

Strabismus, or crossed eyes, is a condition in which the eyes are not properly aligned. One or both eyes may turn in (esotropia), out (exotropia), up (hypertropia), or down (hypotropia). Strabismus is one of the most common ocular conditions diagnosed in children. Prevalence estimates of strabismus vary depending on the country and study methodology, but it affects approximately 2 to 5% of children in the United States [1,2].

Abnormal eye alignment not only impacts proper visual development, but it can also negatively influence the psychosocial well-being of a child. It is well-studied in adults that socially visible strabismus leads to negative social bias causing difficulty with employment procurement, low self-esteem, and altered interpersonal relationships [3,4,5]. The awareness of physical abnormality develops around 6 years old, and once a person is perceived as having an abnormal facial feature, it is almost impossible for him/her to escape the social stigma due to the wide-ranging effects of it [6]. Feelings of embarrassment and social anxiety can be intensified in the adolescent years. In a study from China [7], children with strabismus had a higher prevalence of alcohol use (62.3% vs. 36.3%) and positive screening responses for depression (26.0% vs. 11.6%) and anxiety (10.3 vs. 4.9%).

A substantial body of research has demonstrated that untreated strabismus is a psychosocial stressor. This literature review aims to further investigate the potential association between strabismus and clinically diagnosed psychiatric illness in children.

## 2. Materials and Methods

An electronic database search was performed in Google Scholar and PubMed. In these searches, the following key phrases were used: strabismus, mental illness, mood disorders, psychiatric illness, anxiety, depression, childhood, and adolescence. The latest search was performed on 16 January 2022. Articles that assessed the association between strabismus and common psychiatric diagnoses (attention-deficit/hyperactivity disorder, anxiety disorder, mood disorder, adjustment disorder, bipolar and related disorders, schizophrenia spectrum and other psychotic disorders, substance-related and addictive disorders, obsessive-compulsive disorder, and developmental disorder as defined by the International Classification of Disease, Ninth Revision, Clinical Modification or the International Classification of Disease, Tenth Revision, Clinical Modifications) in children and adolescents were included in the study. Studies that examined mental health problems arising in adulthood in patients with prior strabismus diagnosis in childhood were also selected. Also included were articles that assessed depression and anxiety symptoms based on standardized screening surveys or questionnaires. There were no gender, language, or demographic restrictions. Studies with duplicate data sets were excluded.

## 3. Results

### 3.1. Characteristics of Included Studies

Table 1 provides a description of the included studies. A total of 11 studies was included in this review. The publication dates were between 2006 and 2022. Five studies reported data from the United States and one study each from Denmark, Taiwan, China, Republic of Korea, Israel, and Turkey. Among the studies, five were case–control studies, four were cross-sectional studies, and two were cohort studies.

### 3.2. Association with Attention-Deficit/Hyperactivity Disorder (ADHD)

ADHD is one of the most common mental disorders affecting children [18]. It is characterized by inattention, hyperactivity, and impulsivity. Five studies reported the prevalence of ADHD in children with strabismus. According to one large-scale cohort study with 2049 patients [17], the incidence of ADHD per 1000 person-years was 5.39 in the strabismus group and 3.23 in the control group (*p* < 0.001). Further analysis dividing the strabismus group into those with esotropia and exotropia found the incidence of ADHD for both esotropia (hazard ratio [HR]: 2.04; 95% confidence interval [CI]: 1.36–3.06; *p* < 0.001) and exotropia (HR: 1.44; 95% CI: 1.03–2.03; *p* = 0.038) groups to be significantly greater than controls. Similarly, in a large cross-sectional study with 327,076 subjects [8], the prevalence of ADHD was greater in the strabismus group than in the control group (odds ratio [OR]: 1.36; 95% CI: 1.27–1.45; *p* < 0.001). When comparing the prevalence of ADHD according to strabismus type, the prevalence of ADHD was again significantly greater in both the exotropia (OR: 1.17; 95% CI: 1.10–1.23; *p* < 0.001) and esotropia (OR: 1.20; 95% CI: 1.09–1.33; *p* < 0.001) groups than controls; however, no difference was found between the hypertropia group (OR: 1.33; *p* = 0.079) and controls.

In a case–control study conducted in the United States with 407 subjects [14], Mohney et al. found that children with exotropia were more likely to develop ADHD than controls (*p* = 0.001); however, children with esotropia (*p* = 0.900) were no more likely to develop ADHD than control subjects. In a later study Mohney co-authored where the strabismic cohort included only patients with congenital esotropia, where perhaps the earlier onset of disease exposed children to a more prolonged period of stress, there was still no significant difference in the prevalence of ADHD between children with esotropia and control subjects [15]. It is unclear why exotropia and not esotropia is associated with greater prevalence of ADHD. It is worthy to note that in this study [15], congenital esotropia was found to have an increased risk of subsequent development of overall mental illness. In only one case–control study, Merdler et al. [13] found no significant difference in ADHD prevalence between the strabismus and control groups (*p* = 0.206). However, the authors speculate that perhaps ADHD was underdiagnosed in the strabismus group when patients’ learning disabilities from inattention and hyperactivity were attributed to uncorrected strabismus instead.

### 3.3. Association with Anxiety Disorder

Anxiety disorder involves persistent and excessive worry about a specific activity, object, or situation that interferes with daily life. Seven studies reported on the association of anxiety in youth with strabismus. Lee et al. [11] evaluated 12,005,189 patients in a cross-sectional study and found children with strabismus had a higher prevalence of anxiety (OR: 2.01; 95% CI: 1.99–2.04; *p* < 0.001) than controls. Further analysis dividing the strabismus group into those with esotropia, exotropia, and hypertropia found the incidence of anxiety to be significantly greater in all strabismus groups (*p* < 0.001 for all groups) compared to controls. Similarly, in a large case–control study with 662,641 adolescents, a significant association between strabismus and anxiety disorder was noted (OR: 1.92; 95% CI: 1.02–3.57; *p* = 0.047) [13]. In line with the aforementioned studies, Lin et al. [7] assessed anxiety amongst middle- and high-school-aged children using self-administered questionnaires and found positive responses to screening questions for anxiety were significantly more common among children with strabismus (10.3% vs. 4.9%, *p* < 0.020). In another survey-based study, Cumurcu et al. [9] found greater total scores on the SCARED (Screen for Child Anxiety-Related Emotional Disorders) (*p* < 0.001) and its subscores for social phobia (*p* = 0.004), school phobia (*p* < 0.001), and separation anxiety (*p* = 0.050) in strabismus patients than the control group.

In two separate studies where children with strabismus were found to have significantly increased odds of developing overall mental illness, when analyzing anxiety disorder as an individual mental illness, no significant difference was found [8,15]. In one of these two studies [15], the sample size of patients with anxiety disorder was too small to demonstrate statistical significance. Similarly, Mohney et al. [14] evaluated 407 children in a case–control study and found that children with strabismus, specifically exotropia, were significantly more likely to develop mental illness later on in life; however, when looking specifically at anxiety individually, the sample size was too small to draw an inference. In an article Mohney co-authored 1 year later [12], by extending the original cohort to include patients who were diagnosed with intermittent exotropia during a 10-year to 20-year period, he noted that girls were at greater likelihood of developing anxiety disorder. The same association with anxiety disorder was not noted among boys with intermittent exotropia. The reason for the gender difference is unclear, although it has been reported that females with strabismus have more difficulty securing gainful employment compared with controls [5], whereas the same is not true of males. It may be that the lived experience and social pressure from strabismus is more strongly internalized by girls than boys.

### 3.4. Additional Associations with Mental Illness

In addition to ADHD and anxiety disorder, other psychiatric disorders and their association with strabismus were also studied, though the results were less conclusive. In a large cross-sectional study with 12,005,189 patients, Lee et al. [11] found a moderate association between strabismus and schizophrenia-spectrum disorder (OR: 1.83; 95% CI: 1.76–1.90; *p* < 0.001). In a prospective cohort study, Schiffman et al. [16] examined 265 children between 11 and 13 years old for ocular motility disorder, and two decades later pulled their psychiatric data when they were 31–33 years old. They found that children who later developed schizophrenia-spectrum disorder in adulthood had significantly higher childhood strabismus scores compared to children who later developed other non-schizophrenia psychopathology in adulthood (*p* = 0.013) or children who did not later develop any mental illness (*p* = 0.007). Three smaller cross-sectional studies [8,14,15] also evaluated the association between strabismus and schizophrenia, and while a significant overall prevalence of mental illness with strabismus was noted, when looking specifically at schizophrenia there was no association with strabismus.

Six studies on the association between depression and strabismus in children reported mixed findings. Three studies reported a significant positive correlation [7,11,12], while three reported no significant association [8,14,15]. This discrepancy emphasizes the need for further research in the area.

### 3.5. Impact of Corrective Strabismus Surgery

The improvement in social life and psychological stressors following correction of strabismus is well-acknowledged [19,20,21]. However, limited studies exist on the impact of strabismus correction on the risk of developing mental illness. In a case–control study, Kilgore et al. [10] examined 184 children with intermittent exotropia and found that there was no significant difference in the prevalence of mental illness between the surgical and non-surgical groups (*p* = 0.300). In another case–control study, Merdler et al. [13] also found no association of any of the mental disorders examined (anxiety, mood, adjustment, ADHD) with the status of strabismus correction (*p* = 0.060, *p* = 1.000, *p* = 0.165, and *p* = 0.747, respectively). The results from these two small studies suggest that factors besides psychological stressors predispose children to the later development of mental illness. Additional large-scale studies are needed in this area. It would be useful to capture factors such as the duration of misalignment, timing of intervention, and success of intervention on the development of psychiatric disorders.

## 4. Discussion

The relationship between strabismus and psychiatric illness in children has not historically been a well-understood topic. Only in recent years, where there is growing emphasis on mental health and its early diagnosis and treatment, has the relationship between various ocular and psychiatric phenomena been increasingly well-characterized. While a substantial body of research has demonstrated that strabismus is a psychosocial stressor leading to a decreased quality of life, little is known regarding the relationship between strabismus and the development of mental illness. In this review, we reviewed eleven studies that examined the potential association between strabismus and pediatric psychiatric illness. The results from our review suggest that strabismus is associated with increased odds of mental illness in children, particularly ADHD and anxiety disorder. Esotropia and exotropia are both more frequently associated with mental illness than vertical strabismus, though this is likely related to the greater prevalence of horizontal strabismus in the pediatric population.

Speculation regarding the mechanisms responsible for the prevalence of mental illness in strabismus patients is rare and poorly understood. Mohney et al. [14] hypothesized in 2008 that the association may be hereditary in nature and result from interactions between multiple susceptible genes. In the literature, there is at least one form of strabismus, constant exotropia, that has been shown to be genetically linked with schizophrenia through a single-gene mutation [22]. Later in 2012, Olson et al. [15] reported increased odds of developing mental illness in patients with congenital esotropia. In this same study, they noted an increased prevalence of prematurity and difficulty at the time of delivery in strabismic patients than in controls. It may be possible that prenatal stressors lead to neurobehavioral changes in development and an increased risk of psychiatric illness.

It is most likely that the relationship between strabismus and mental illness is a complex one of premorbid genetic predisposition along with psychosocial risk factors in early childhood. In adults, having socially significant strabismus leads to negative social bias, difficulty with employment procurement, low self-esteem, and altered interpersonal relationships [3,4,5]. In children, these feelings of low self-esteem and rejection may be a handicap to educational success and peer socialization. Most childhood strabismus have an onset before the age of 10 when the emotional maturity of a child is still developing. The lived experience and everyday stress living with strabismus (altered social interactions, frequent doctor’s appointments, self-doubt, etc.) likely have an additive effect over time and contribute to the development of certain psychiatric illnesses, particularly mood disorders such as anxiety and depression.

There are several limitations to our review. First, the majority of our studies relied on retrospective or cross-sectional reports, and as such causality between strabismus and mental illness cannot be established. Second, the limited follow-up period of the strabismic patients through young adulthood in the studies may underappreciate the prevalence of psychiatric illnesses that have a later onset in the second and third decades of life in this cohort. Lastly, the low sample size of patients with strabismus with each individual mental illness limited individual analyses.

In conclusion, our review highlighted the increased prevalence of mental illness in children with strabismus. These results should alert both ophthalmologists and pediatricians to have a heightened awareness of the psychosocial burden that may affect children with strabismus and consider early mental health screening and referral as needed.

## Figures and Tables

**Table 1:** Description of included studies.

Author (Year)	Region, Country	Study Period	Study Design	Sample Size	Main Findings
Choi (2022) [8]	Republic of Korea	2011–2017	cross-sectional	327,076 children with strabismus, 327,076 controls (sex-matched and age-matched within 5-year age range)	Pediatric mental illness was more likely to develop in strabismus group than the control group (*p* < 0.001). The prevalence of developmental disorder, autism, ADHD, and pediatric behavioral and emotional disorders was significantly greater in the strabismus group than control group (*p* < 0.001 for all).
Cumurcu (2015) [9]	Malatya, Turkey	Not reported	cross-sectional	42 children with strabismus, 47 control subjects	The total scores of SCARED and its subscores for separation anxiety, social phobia, and school phobia were significantly higher in the strabismus group than control (*p* = 0.004, *p* = 0.004, *p* < 0.001, *p* = 0.050, respectively).
Kilgore (2014) [10]	Olmsted County, Minnesota	1975–1994	case–control	184 children with IXT (*n* = 63 children underwent strabismus surgery; *n* = 121 children did not undergo surgery)	Success of strabismus surgery (<10 prism diopters) was not associated with a decreased occurrence of mental illness (*p* = 0.300).
Lee (2022) [11]	USA	January 2007–December 2017	cross-sectional	352,636 children with strabismus, 11,652,553 control subjects	Children with strabismus have a higher prevalence of mental illness diagnosis—OR were 2.01 for anxiety disorder, 1.83 for schizophrenia, 1.64 for bipolar disorder, and 1.61 for depressive disorder (*p* < 0.001 for all).
Lin (2014) [7]	Guangdong, China	2009–2010	cross-sectional	292 children with strabismus, 3611 control subjects	History of alcohol use (62.3% vs. 36.3%) and positive screening responses for depression (26% vs. 11.6%) and anxiety (10.3% vs. 4.9%) were significantly more common among children with strabismus (*p* < 0.010 for all) than control subjects.
McKenzie (2009) [12]	Olmsted County, Minnesota	1975–1994	case–control	183 children with IXT, 183 control subjects (age- and sex-matched)	Children with IXT were 2.7 times more likely to develop a psychiatric illness than controls (*p* < 0.001). Rates of mental illness among patients with IXT were significantly higher in males than females (63% vs. 33%, *p* < 0.001). Males with IXT were more likely to develop depression (*p* = 0.020) and adjustment disorder (*p* = 0.020) compared with their controls. Females with IXT were more likely to develop ADHD (*p* = 0.007), anxiety or phobia (*p* = 0.020), and learning disability (*p* = 0.040).
Merdler (2017) [13]	Israel	2005–2013	case–control	1959 adolescents with strabismus, 661,043 control subjects	There was a significant association between strabismus and anxiety disorder (OR = 1.91; *p* = 0.047). No significant association between status of strabismus correction and anxiety disorder, mood disorder, adjustment disorder, or ADHD (*p* = 0.060, *p* = 1.000, *p* = 0.165, and *p* = 0.747, respectively).
Mohney (2008) [14]	Rochester, Minnesota	January 1985–December 1994	case–control	407 children with strabismus, 407 controls (age- and sex- matched)	Children with exotropia were 3.1 times more likely to develop a psychiatric disorder than control subjects (*p* < 0.001). Children with esotropia were no more likely to develop mental illness than control subjects (*p* = 0.650).
Olson (2012) [15]	Olmsted County, Minnesota	January 1965–December 1994	case–control	127 children with congenital esotropia, 127 controls (age- and sex-matched)	Individuals with congenital esotropia were 2.6 times more likely to be diagnosed with a mental illness by early adulthood than control subjects (*p* = 0.019).
Schiffman (2006) [16]	Copenhagen, Denmark	1972–1992	cohort study	265 total patients (*n* = 90 children with at least one parent with schizophrenia; *n* = 93 children with at least one parent with non-schizophrenia psychiatric diagnosis; *n* = 82 children with no parental diagnosis of mental illness)	Children who later developed schizophrenia-spectrum disorder had significantly higher strabismus scores compared to children who later developed other non-schizophrenia psychopathology (*p* = 0.013) or children who did not later develop a mental illness (*p* = 0.007). The strabismus score did not significantly differ between offspring of parents with schizophrenia, offspring of parents with other non-schizophrenia psychotic disorder, and offspring of parents with no mental illness.
Tsai (2021) [17]	Taiwan	2000–2010	cohort study	2049 children with strabismus, 8196 controls (age- and sex-matched)	Greater incidence of ADHD per 1000 person-years in strabismus group than control group (5.39 vs. 3.23; *p* < 0.001).

SCARED: The Screen for Child Anxiety-Related Emotional Disorders; IXT: intermittent exotropia; OR: odds ratio; ADHD: attention-deficit/hyperactivity disorder.

## Data Availability

Not applicable.
